# Typhim vi immunization assists to discriminate primary antibody responses in hematological malignancies

**DOI:** 10.1016/j.mex.2020.100936

**Published:** 2020-05-29

**Authors:** J. Ochoa-Grullón, C. Orte, A. Rodríguez de la Peña, K. Guevara-Hoyer, G. Cordero Torres, M. Fernández-Arquero, I. Serrano-García, M.J. Recio, R. Pérez de Diego, S. Sánchez-Ramón

**Affiliations:** a Department of Immunology, IML and IdSSC, Hospital Clínico San Carlos, Madrid, Spain; b Department of Immunology, Ophthalmology and ENT, School of Medicine, Complutense University School of Medicine, Madrid, Spain; c Immunodeficiency Interdepartmental Group (GIID), Madrid, Spain; d Laboratory of Immunogenetics of Human Diseases, IdiPAZ Institute for Health Research, Madrid, Spain; e Department of Epidemiology and Preventive Medicine, Hospital Clínico San Carlos, Madrid, Spain

**Keywords:** Primary responses, secondary responses, specific polysaccharide Ab response, Pneumo 23, Typhim Vi, Hematological Malignancies, ROC curve, Youden index, Union index, cut-off value

## Abstract

Assessment of specific antibody (Ab) production to polysaccharide antigens is clinically relevant, identifying patients at risk for infection by encapsulated bacteria and thus enabling a more rigorous selection of patients that can benefit of immunoglobulin replacement therapy. Classically, the gold-standard test is the measurement of antibody production to pure polysaccharide pneumococcal (PPV) immunization. Several factors, including introduction of conjugate vaccination schedule, serotyping analysis, high baseline Ab levels, have hindered the evaluation of polysaccharide antigens. This is even more difficult in secondary immunodeficiencies (SID), where patients can show secondary responses despite lack of primary antibody responses and present with recurrent or severe infections. Assessment of specific Ab production to pure S*almonella typhi Vi* polysaccharide (TV) immunization has been proposed as a complementary test to PPV, given its low seroprevalence. To set the optimal cut-off value for PPV and TV response in SID, we tested different biostatistical methodologies, including ROC analysis, Youden index, Union index and Closest-topleft in a cohort of 42 SID patients and 24 healthy controls. The statistically chosen cut-offs value pre-post TV Ab ratio was ≥5, (sensitivity of 90%, specificity of 100%) and a postvaccination TV concentration of 28.5 U/mL (sensitivity of 90%, specificity of 95%), showing relevant clinical correlate.

## Highlights

• *Typhim Vi response have been proposed as a new strategy for the assessment of specific polysaccharide antibody response in SID due to HM.*
• *Different biostatistical methodologies may stablish the best cut-off value to discriminate Typhim Vi response.*
• *Typhim Vi IgG responses may better discriminate primary Ab responses showing relevant clinical correlate.*

## Graphical Abstract

**Figure:**
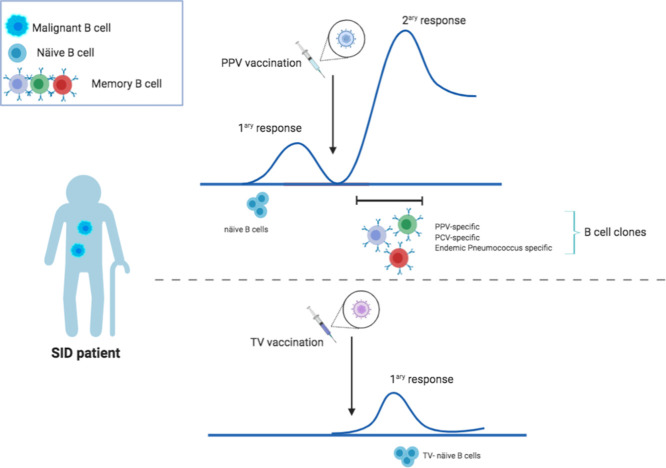

Specifications table

**Table:** 

Subject Area:	Immunology and Microbiology
More specific subject area:	*Secondary Immunodeficiencies in Hematological Malignancies.*
Method name:	*Typhim Vi Ab response for the diagnosis of defective primary responses in SID.*
Name and reference of original method:	• B.L. Ferry, S.A. Misbah, P. Stephens, Z. Sherrell, H. Lythgoe, E. Bateman, C. Banner, J. Jones, N. Groome, H.M. Chapel, Development of an anti-Salmonella typhi Vi ELISA: assessment of immunocompetence in healthy donors, Clin. Exp. Immunol. 136 (2004) 297–303. https://doi.org/10.1111/j.1365-2249.2004.02439.x. • S. Sánchez-Ramón, J. de Gracia, Am. García-Alonso, J.J. Rodríguez Molina, J. Melero, A. de Andrés, J.M. García Ruiz de Morales, A. Ferreira, J.G. Ocejo-Vinyals, J.J. Cid, J.M. García Martínez, T. Lasheras, M.L. Vargas, J. Gil-Herrera, M.C. García Rodríguez, J.L. Castañer, L.I. González Granado, L.M. Allende, P. Soler-Palacin, L. Herráiz, M. López Hoyos, J.M. Bellón, G. Silva, D.M. Gurbindo, J. Carbone, C. Rodríguez-Sáinz, N. Matamoros, A.R. Parker, E. Fernández-Cruz, Multicenter study for the evaluation of the antibody response against salmonella typhi Vi vaccination (EMPATHY) for the diagnosis of Anti -polysaccharide antibody production deficiency in patients with primary immunodeficiency, Clin. Immunol. 169 (2016) 80–84. https://doi.org/10.1016/j.clim.2016.05.006. • M.T. Bausch-Jurken, J.W. Verbsky, K.A. Gonzaga, N.P. Elms, M.K. Hintermeyer, S.B. Gauld, J.M. Routes, The Use of Salmonella Typhim Vaccine to Diagnose Antibody Deficiency, J. Clin. Immunol. 37 (2017) 427–433. https://doi.org/10.1007/s10875-017-0406-6. • H. Schaballie, B. Bosch, R. Schrijvers, M. Proesmans, K. De Boeck, M.N. Boon, F. Vermeulen, N. Lorent, D. Dillaerts, G. Frans, L. Moens, I. Derdelinckx, W. Peetermans, B. Kantsø, C.S. Jørgensen, M.-P. Emonds, X. Bossuyt, I. Meyts, Fifth Percentile Cutoff Values for Antipneumococcal Polysaccharide and Anti-Salmonella typhi Vi IgG Describe a Normal Polysaccharide Response, Front. Immunol. 8 (2017). https://doi.org/10.3389/fimmu.2017.00546. • J. Kumarage, S.L. Seneviratne, V. Senaratne, A. Fernando, K. Gunasekera, B. Gunasena, P. Gurugama, S. Peiris, A.R. Parker, S. Harding, N.R. de Silva, The response to Typhi Vi vaccination is compromised in individuals with primary immunodeficiency, Heliyon. 3 (2017) e00333. https://doi.org/10.1016/j.heliyon.2017.e00333. • I. Unal, Defining an Optimal Cut-Point Value in ROC Analysis: An Alternative Approach, Comput. Math. Methods Med. 2017 (2017) 1–14. https://doi.org/10.1155/2017/3762651.
Resource availability:	*NA*

## Introduction

Impairment of specific antibody (Ab) responses characterizes a wide number of primary (PID) and secondary (SID) immunodeficiencies, rendering individuals susceptible to recurrent and severe infections, mainly by encapsulated bacteria as well as rare opportunistic pathogens [1], [2], [3]. Assessment of specific Ab responses is hence clinically relevant and enable a more rigorous selection of patients that can benefit of immunoglobulin replacement therapy (IgRT).

A growing population of specific Ab defects in the last years are patients with B cell haematological malignancies (HM), which show humoral and cell-mediated defects due to the underlying disease and to specific B-cell targeted therapies (BCTT) impact [4]. Moreover, recurrent or severe infections are a major cause of morbidity and mortality in HM [5], [6], [7].

Diagnostic assessment of humoral T-cell independent responses is classically performed by measuring Ab responses to polysaccharide antigens, such as 23-valent pneumococcal polysaccharide vaccine (PPV), meningococcal, isohaemagglutinins and more recently *Salmonella typhi* Vi polysaccharide vaccine (TV). The latter has been suggested as a complementary approach when interpretation of gold standard test (PPV) might be challenging [8], [9], [10], [11], [12].

The currently accepted consensus criteria for specific anti-PPV IgG responses is the serotype-specific enzyme-linked immunosorbent assay (ELISA) validated by the World Health Organization (WHO). Given that each serotype requires a single ELISA, the serotype-specific assay (SSA) is too expensive and time-consuming to be used in routine clinical practice. As a result, it has not been widely disseminated and is available in only a few highly qualified laboratories. In PID, a global PPV ELISA has shown very good correlation with serotypes when IgG is below 4 mg/dL, while requires SSA when >4 mg/dL [13]. Other alternative approaches have also been suggested, such as in house multiplex [14].

Although different assay protocols may be acceptable, one of the main concerns is still PPV Ab response interpretation. A differential feature of SID with respect to PID is that HM patients may show high baseline levels of PPV responses while lacking primary Ab responses to neoantigens. In a way to overcome those difficulties, the present study was designed to assess the value of the Ab response of Typhim Vi compared to PPV responses in order to discriminate primary and secondary Ab responses in previously immunocompetent patients, a feature that is particularly useful in SID patients.

## Material and methods

### Subjects

An observational study was conducted at the Hospital Clínico San Carlos of Madrid, Spain, between 2013 and 2015. The study population was composed of 42 adult patients (aged 25 to 86 years; 27 females) diagnosed with HM based on the WHO Classification of Tumours of Haematopoietic and Lymphoid Tissue [15]. Most of the patients were referred to immunological evaluation due to recurrent or severe infections. Twenty-four asymptomatic volunteers of similar age and gender to the patient group were selected as the healthy control group (HC-Group). To our knowledge, these individuals had not received Typhim Vi vaccination, and thus these samples were used to determine baseline concentrations of Typhi Vi IgG antibodies.

All the procedures were approved by the Ethical Committee of the center. None of the patients had previously received IgRT before nor previous pneumococcal conjugated vaccine in the last 3-5 years.

### Serum collection and Ab testing

In order to compare the two polysaccharide vaccines, all subjects received the gold-standard 23-valent pneumococcal polysaccharide vaccine (PPV) (Pneumo 23®, Sanofi Pasteur MSD Limited, Maidenhead, Berks, United Kingdom), and Typhim Vi polysaccharide vaccine (TV) (Typhim Vi^TM^, Sanofi Pasteur MSD). Blood samples were obtained via venopuncture on day 0 prior to vaccination and on day 30±3 after vaccination. Vaccines were administered by in- tramuscular injection at the same visit in two distinct sites (right and left deltoid muscle). Pre- and post-vaccination blood was separated by centrifugation and serum was stored at -40° C until simultaneous performance of specific IgG Ab tests.

### Anti-Polysaccharide IgG ELISA

PPV and Typhim Vi specific IgG Ab response were measured for 66 subjects using global ELISA commercial kits; VaccZyme^TM^ anti-PCP IgG and VaccZyme^TM^ anti-*S. typhi* Vi human IgG (The Binding Site Group Ltd, Birmingham, United Kingdom) and were analyzed on a Triturus analyzer (Grifols S.A., Barcelona, Spain). The anti-PCP IgG assay is composed of a mixture of 23 pneumococcal serotypes: 1-5, 6B, 7F, 9N, 9V, 10A, 11A, 12F, 14, 15B, 17F, 18C, 19F, 19A, 20, 22F, 23F, and 33F. Serum samples pre- and post-vaccination were collected for all patients and controls. We previously demonstrated high reliability of the ELISA assay in our laboratory (*data not published*). Microwells were pre-coated with the Pneumococcal Capsular Polysaccharide (PCP) or with Typhi Vi antigen depending on the immunoassay kit. Calibrators and controls were pre-adsorbed against capsular polysaccharide and samples are diluted (1:100). Subsequent, 100 µL of each calibrators, controls and diluted patient were added to the wells. Antibodies recognizing the antigen (PCP or Typhi Vi, depending on the precoated well) bind during the first incubation. After washing the wells (x3) with 250-350 µL of wash buffer (to remove all bound proteins), peroxidases labelled rabbit anti-human IgG conjugate were added (100 µL). The conjugate binds to the captured human antibody and the excess unbound conjugate was removed by a further wash step. The bound conjugate is visualized with 3,3′,5,5′ tetramethylbenzidine (TMB) substrate which gives a blue reaction, proportional to the concentration antibody in the sample. Phosphoric acid were added to stop the reaction. Pre- and post-vaccination samples were run in duplicate on the same ELISA plates according to manufacturer's instructions to minimize intraassay variability and for assay validation.

### Immunoglobulins’ analysis

Total immunoglobulins, IgG subclasses and C4 complement factor were measured using commercial kits on an Optilite nephelometer (The Binding Site Group Ltd, Birmingham, UK).

### Measurement of T-, B- and NK- lymphocytes

EDTA anticoagulated peripheral blood samples were tested by lyse-no-wash method on routinely calibrated FACSCanto II flow cytometer (Becton-Dickinson, San Jose, CA) using certified BD Multitest TBNK kit and data analysed using BD FACS software version 8.0. Each tube was run until 10, 000 events were recorded or until the tube was exhausted. The reports contained CD4^+^and CD8^+^ T-lymphocytes, B and NK cell percentages and absolute counts expressed as % of total lymphocytes and absolute counts (cells/mm^3^). Reference values for CD4^+^ T-lymphocytes: 51-66%; 464-1,721/µL. CD8^+^ T-lymphocytes: 28-36%; 178-853/µL. CD19 B lymphocytes: 7-13%; 92-515/µL; NK cells CD3-CD56^+^CD16^+^: 8-19%; 82-594/µL.

### Specific antibody responses after immunization

Specific Ab concentrations were reported for PPV IgG as mg/dL (range, 0.33-27 mg/dL) and TV IgG as U/mL (range, 7.4-600 U/mL). Samples resulting in a value below the lower limit concentration detected in the standard curve (<7.4 U/mL) were set randomly to 0-7 U/mL for data analysis [16]. The values of these responses are also given as the ratio between pre- and post- immunization antibody levels. Responders were defined as individuals obtaining a fold increase (FI) ≥3 for PPV IgG and TV IgG ≥5 according to ideal cut-off level previously proposed for this patients’ population [12].

### Statistical analysis

Descriptive data are presented as mean±standard deviation (SD) or median values (interquartilic range, IQR). Differences in Ab responses between groups were evaluated using a Mann-Whitney U test for continuous variables, and the chi-squared test or Fisher´s exact test for categorical variables, as appropriate. Receiver operating characteristic (ROC) curves were plotted and the area under the curves (AUCs) was calculated for pre- and post-immunization antibody titers before and after vaccination. The best cut-off values were chosen according to: (1) ROC curves, (2) Youden index calculation, (3) Closet topleft and (4) Index of Union, as defined the most adequate statistical method to set cut-off levels. For each identified cut-off value, positive (PosPV) and negative predictive value (NegPV) were calculated. Statistics were analysed with SPSS (Chicago, Illinois) and GraphPad Prism software (GraphPad Software, La Jolla, CA, USA version 8), p<0.05 was considered statistically significant.

## Results

### Hematological status and classification

HM patients were classified as: non-Hodgkin lymphoma (NHL) (n=17, 40%), monoclonal gammopathy of undetermined significance (MGUS) (n=13, 31%), chronic lymphocytic leukaemia (CLL) (n=10, 24%), Hodgkin lymphoma (HL) (n=1, 2%) and multiple myeloma (MM) (n=1, 2%).

None of the patients were receiving chemotherapy during the immunological evaluation period. Previous chemotherapy regimens included: R-CHOP, FCR, ABVD, Melphalan and Dexametasone. Excluding the MGUS paraprotein subgroup, 21 patients (72%) had received one cycle chemotherapy (4-6 weeks), 6 patients (21%) at least two cycles and 2 patients (7%) were in a “watch and wait” phase. Mean follow-up duration since study inclusion was 26±15 months (median, 24 months). During the follow-up period, the HM remained stable in 40 patients (95%) and worsened in 2 patients (5%). Two patients (5%) died, one patient due to pneumonia and other patient due to HM progression. Six patients (14%) of the NHL group had received stem cell transplant and two patients (5%) of the CLL group were splenectomised secondary to refractory autoimmune haemolytic anaemia (AHAI). Table 1 displays immunological assessment previous to polysaccharide vaccination. Different from what was expected, the NHL group showed a severe immune defect in possible association with more aggressive chemotherapy regimen.

**Table 1:** Immunological assessment per group previous polysaccharide vaccination

	NHL No: 17	MGUS No: 13	CLL No: 10
**IgG (mg/dL)**	424±303 356 (530)	1.360±793 1.131(1.106)	553±358 344 (608)
**IgA (mg/dL)**	48±61 18 (81)	141±92 111 (146)	96±155 22 (68)
**IgM (mg/dL)**	42±45 25 (67)	252±385 95 (169)	29±31 12 (37)
**IgG1 (mg/dL)**	268±223 194 (356)	844±607 523 (732)	280±150 240 (233)
**IgG2 (mg/dL)**	130±100 95 (115)	286±193 235 (235)	228±197 118 (328)
**%Neutrophils**	51±12 56 (12)	49±18 51 (25)	25±19 14 (45)
**Neutrophils (cells/mm^3^)**	2,403±1.463 2,600 (2,100)	2,057±1.341 3.050 (2.450)	1,836±1.704 2,800 (2,400)
**Lymphocytes (cells/mm^3^)**	1,611±931 1,400 (931)	1,772±1,401 1,700 (1,500)	11,800±16,881 4,900 (9,850)
**%B lymphocytes**	7±6 6 (9)	13±12 11 (9)	49±35 45 (76)
**CD19 B cell lymphocytes (cells/mm^3^)**	107±143 62 (152)	142±82 145 (142)	2,888±3,486 1,251 (5,235)
**%CD4^+^ T-lymphocytes**	29±7 26 (11)	42±12 44 (21)	19±16 16 (31)
**CD4^+^ T-lymphocytes (cells/mm^3^)**	464±295 434 (562)	751±362 754 (496)	837±437 574 (909)
**%NK cells**	16±9 14 (17)	12±6 11 (9)	7±7 4 (9)
**NK cells (cells/mm^3^)**	202±156 121 (252)	207±132 146 (200)	473±404 204 (755)

Data are presented as mean±standard deviation; median (Interquantilic Range; IQR).

M-component in the MGUS group (%; g/dL and Kappa/Lambda ratio): 13.8±7.71; 15.3 (12.8%). 1.03±0.63; 1.09 (1.03 g/dL). 0.99±1.15; 1.02 (2).

Reference values: serum immunoglobulins (mg/dL) IgG: 767–1,590; IgA: 61–356; IgM 37–286.

Immunoglobulin subclasses (mg/dL) IgG1: 341–894; IgG2: 171–632.

Reference values for CD4+ T-lymphocytes: 51-66%; 464-1,721. CD8+ T-lymphocytes: 28-36%; 178-853. CD19 B lymphocytes: 7-13%; 92-515; NK cells CD3-CD56+CD16+: 8-19%; 82-594.

### Assay validation for ELISA test

Mean concentration and percentage of coefficient of variation (%CV) were calculated for each sample. The %CV for internal quality certificate (iQC) was 1.2% and 3.2% for high and low PCP Ab levels; and 2.3% for high and 6.4% for low Typhim Vi Ab levels. Analyses were repeated when the coefficient of variation between duplicates was above 15% according to manufacturer instructions, which was not the case.

### Baseline specific antibody concentrations

Contrary to baseline PPV Ab with 76% (32 out of 42) above 4.4 mg/dL of HM patients, only 7% (3 out of 42) showed baseline concentrations of TV IgG Ab above the cut-off of 7.4 U/mL (p<0.001). In the HC-Group, 18 out of 24 (75%) had baseline Ab concentrations for PPV *versus* 7 of 24 (30%) for TV, disclosing the low seroprevalence of *S. Typhi* in both groups, compatible with previous reports.

### Primary *versus* secondary polysaccharide antibody responses

**Fig. 1:**
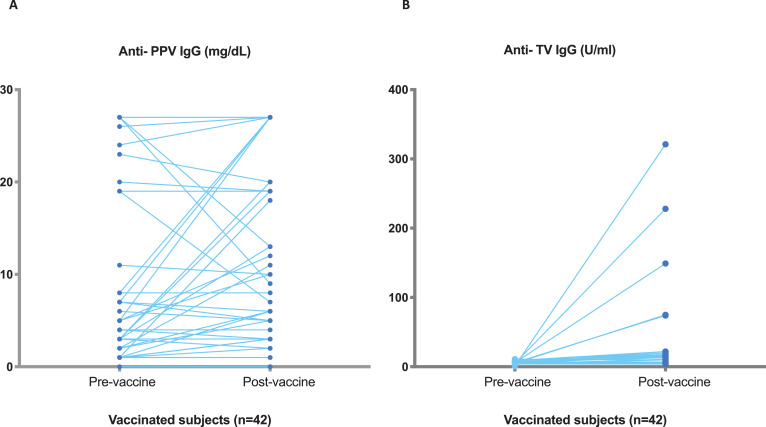
Antibody concentrations Pre- Post TV IgG and PPV IgG in HM patients referred for immunological investigation. **(A)** Pre- Post PPV responses, **(B)** Pre- Post TV responses.

In the HC-group, the median post vaccination concentration of specific Ab after immunization was 88 U/mL (range 34–600 U/mL) for TV and 27 mg/dL (7.5-27 mg/dL) for PPV. Using the minimum post vaccination concentration for TV IgG in HC (34 U/mL) as a cut-off, 5 of 42 HM-Group achieved a post vaccination >34 U/mL. The tendency in PPV response between pre- and post-vaccination in these 42 patients was clearly different than that obtained for TV, as expected. Fig. 1 shows raw data pre-post antibody titres for both antigenic challenges. Indeed, using the minimum post vaccination for PPV (7.5 mg/dL), 11 of 42 achieved a post vaccination >7.5 mg/dL, suggesting PPV secondary responses in previously exposed patients. Three out of this 11 (27%) had baseline Ab PPV concentrations above 4.4 mg/dL and none of them response for TV.

**Fig. 2:**
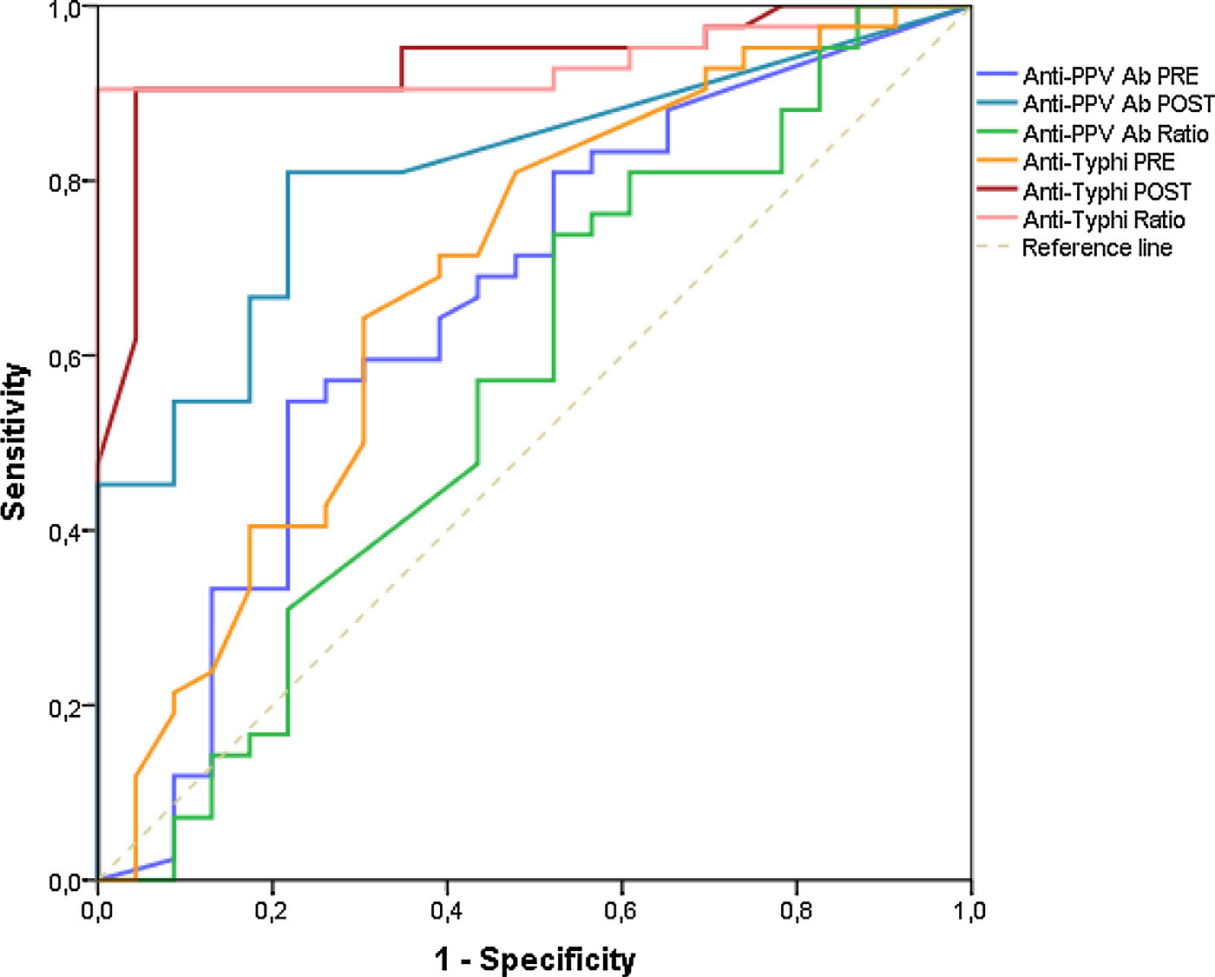
ROC curve

Based on previous ROC analysis to determine the optimal threshold for vaccination response in SID with respect to healthy controls, the statistically chosen cut-off value was a 5-FI for TV IgG, with diagnostic sensitivity of 91%, specificity of 100%, and positive predictive value (PosPV) of 97%; and 3-FI for PPV, with diagnostic sensitivity of 73%, specificity of 47%, and PosPV of 72% **(**Fig. 2**)** according to this cut-off.

To validate statistically the diagnostic performance of the ROC curve analysis, other biostatistical methods were tested **(**Table 2**)**, such as Youden index calculation and the union index, both developed to minimize misclassification rate. Based on the calculation on Youden index and Closet topleft, the best threshold for TV pre-post Ab ratio was ≥5, achieving a diagnostic sensitivity of 91% and specificity of 100%. The cut-off level for post-TV immunization was 28.5 U/mL, with diagnostic sensitivity of 90% and specificity of 95%. Only 5 out of 42 (12%) of the HM-Group achieved a 5-FI and a post-vaccination concentration >28.5 U/mL for TV IgG *versus* 24 of 24 (100%) in the HC-Group.

**Table 2:** Biostatistical methods to define PPV and Typhim Vi cut-off levels.

Criterion	AUC (95% CI)	Se	Sp	PosPV	NegPV	P-value	Youden index cut-off	Union index cut-off	Closest-topleft cut-off
**PRE anti-PPV Ab**	0.65 (0.51-0.80)	0.5476190	0.78	0.82	0.48	0.034	4.61	6.95	5.11
**POST anti-PPV Ab**	0.79 (0.69-0.90)	0.8095238	0.75	0.85	0.69	0.054	20.15	19.6	20.15
**POST/PRE anti-PPV Ratio**	0.56 (0.41-0.72)	0.7380952	0.47	0.72	0.51	0.369	3.31	1.14	3.31
**PRE anti-Typhi Ab**	0.68 (0.54-0.83)	0.6428571	0.69	0.79	0.51	0.013	6.30	6.30	6.30
**POST anti-Typhi Ab**	0.93 (0.87-0.99)	0.9047619	0.95	0.97	0.85	<0.001	28.55	44.4	28.55
**POST/PRE anti-Typhi ratio**	0.93 (0.87-0.99)	0.9047619	1.00	1.00	0.85	<0.001	5.11	5.94	5.11

AUC: area under the curve; Se: sensitivity; Sp: specificity; PosPV: positive predictive value; NegPV: negative predictive value.

### Polysaccharide Ab response and clinical correlate

Patients were classified into two groups based on whether or not they were given IgRT according to immunization responses and history of infections: Group IgRT (n=28) and Group non-IgRT (n=14). Within the Group IgRT, 17 patients did not achieve an adequate polysaccharide antibody response neither to PPV nor to TV. An interesting observation was that 83% of HM patients that responded to PPV (10/12) required IgRT, whereas only 1 patient out of 5 (20%) that responded to TV required IgRT (due to concomitant pulmonary lymphagiomatosis diagnosis). The remaining 14 HM patients in the Group Non-IgRT the decision not to treat with IgRT was due to several reasons: patient decision (2); loss of follow-up (2); the severity of infections did not currently justify IgRT decision; and patients on prophylactic antibiotic and follow-up (4). In the Group Non-IgRT, 4 patients responded to TV and 2 to PPV.

Interestingly, 8 out of 42 (19%) patients presented with at least one episode of herpes zoster infection in the last year, of which 63% (5/8) of these patients were associated with TV Ab deficiency.

### IgG and IgM PPV response during plasmablast flux

We performed specific IgM PPV responses in 11 patients in which serum pre-post PPV vaccination was available. One patient out of 11 (9%) presented a 4-fold PPV increase, the same patient also presented an adequate response to IgG PPV response and 5-FI increase to TV. The remaining 10 patients (90%) showed no increase of IgM PPV, although 2 of them had responded by above 3-fold IgG PPV increase, none to TV.

## Discussion

Defining the optimal cut-off for specific Ab responses is important both for diagnostic and therapeutic purposes. ROC curve defines the optimal cut-off as the point maximizing the product of sensitivity and specificity when a continuous variable is considered as a diagnostic biomarker. A biomarker's cut-off level is meaningful for the clinicians when it is clinically interpretable and understandable [17]. Interestingly, based on the analyses of different biostatistical methodologies to set cut-off levels, our results show that 5-fold increase in TV responses and post-immunization TV concentration of 28.5 U/mL best discriminate SID patients with recurrent or severe infections. TV responses could be particularly useful in SID patients, which were previous immunocompetent, and in which PPV may show normal secondary responses but lack primary responses and present with recurrent or severe infections.

Several publications has proposed fold increase (FI) ratio as the most significant parameter to differentiate infectious risk between patients and healthy controls. However, given the variability of TV baseline levels (and the lack of detection below 7 U/mL), post-vaccination concentrations together with FI in antibody production after immunization may be essential for identifying humoral immunodeficiencies. Combined interpretation of both parameters underlines its clinical utility in SID, and thus may result more accurate for selection of SID patients that may benefit from IgRT. In 2017, López et al. published for the first time significantly greater AUCs [Sp 100% (95% CI, 83.2–100)] for the post-immunization titer and the FI increase in overall pneumococcal assay in PID patients, suggesting that post-immunization titer below or equal to 11 mg/dL would unequivocally identify poor responders and it might be useful as a first intention tool. In our cohort, 3 out of 12 (25%) PPV responders did not achieve a post-immunization titer above 11 mg/dL, two of them on IgRT treatment due to recurrent respiratory infections. According to our biostatistical results, we found a PPV post-vaccination threshold of 19.6 mg/dL, yielding a specificity of 75%. Only two out of seven patients (29%) achieved a 3-FI post vaccination, all patients exhibited baseline Ab PPV concentrations above 4.4 mg/dL. On the other hand, regarding Typhim Vi post-immunization titers, we found that a post-immunization threshold of 28.5 U/mL yielded the greatest specificity of 95% and correctly identified responders versus non responders, with complete correlation with FI in our cohort, all TV responders showed ≥5-FI and post-immunization titers above 28.5 U/mL. Other studies had reported similar median post-vaccination values of 21 U/mL and 32 U/mL [8,10].

In the 1980s, only the purified polysaccharide, 23-valent vaccine (PPV23), was available for immunization and subsequent evaluation of immunity [18]. However, controversy on the definition, arbitrary cut-off criteria, assessment and correlation between different methods for the determination of PPV antibodies complicates the assessment of the T-cell independent Ab response. These controversies could be explained by diverse factors: immunogenicity of different PPV serotypes, combined immunological response, age dependent immunogenicity, booster immunization and immunological memory. A common confounding factor is infection caused by *Streptococcus pneumoniae*, which remain the most prevalent cause of infection in HM patients. Response to re-exposure antigen that has previously evoked an immunological response is characterized by the production of IgG antibody response and low IgM, hence PPV does not seen to be useful or reliable for primary responses in HM patients. Since 2004, the almost universal adoption of conjugate pneumococcal vaccines for children and risk groups’ adults may result in withdrawal of pure carbohydrate form of the vaccine [8].

Recent studies have made an enormous effort to establish cut-off values and alternative strategies for an adequate evaluation of T-cell independent humoral response [19], [20], [21]. Our group and others [11,^22^,23] have proposed the Ab production to pure polysaccharide Vi from *Salmonella Typhi* as a complementary biomarker that allows to assess more accurately Ab response in patients with preserved immunological memory, either because they had previously been vaccinated with a pneumococcal conjugate vaccine (T-dependent response) or because previous immune competence (past infection or nasopharyngeal carriers of pneumococcus). This is in contrast to PID patients, in which TV and PPV show acceptable concordance and give complementary information [8,^10^,23].

In our cohort of HM patients, as expected in non-endemic countries, most of the subjects had undetectable pre-vaccination anti-S-typhi Vi IgG antibodies (93%), also valuable in patients receiving IgRT. Vaccination with Typhim Vi vaccines induces a predominantly IgG2 antibody response [24] while does not induce long term immunological memory [25]. To date, all published data on the evaluation of TV IgG response and PPV are based on the comparison of SSA and TV IgG response, which does not reflect the real scenario of routine clinical practice in our country. Results in the normal range for ELISA global test for PPV can be due to antibodies to most or all included serotypes or by high antibodies to only one, for which SSA would be more informative. Despite the small sample size of our cohort of HM patients, our results highlight the relevance of testing primary and secondary Ab responses by TV and PPV immunization in HM patients with infections.

When a diagnostic test is used in any clinical condition, there may be an opportunity to improve the cut-off values with further fine-tuning according to demographical and biological conditions that may be different from a given population for which the test was developed. Most clinical tests fall short of this ideal because of incorrect optimal cut-off values predicted from a specified test [26,27]. A correct assessment of a patient's specific antibody-mediated immunity requires consideration of the patient's age and immunization status before evaluation. Different laboratories should establish their own ranges of antibody concentrations. It may also be informative of humoral reconstitution in individuals receiving IgRT, which is other distinctive feature of SID [12].

In conclusion, our results provide a statistically valid cut-off level for functional Ab responses in patients with SID to hematological malignancy. Hence, TV IgG responses may add value to functional T-cell independent responses in SID HM patients, better discriminating primary Ab responses with relevant clinical correlate, always taking into consideration that IgRT decision should always be individualized.

## Author contributions

JOG and SSR contributed conception and design of the study. AR, RP have contributed to the immunological study. CO and KGH contributed to the database. IS performed the statistical analysis. JOG wrote the first draft of the manuscript, SSR wrote sections of the manuscript and SSR reviewed the manuscript. All authors contributed to manuscript revision, read and approved the submitted version.

## Declaration of Competing Interest

The authors declare that they have no known competing financial interests or personal relationships that could have appeared to influence the work reported in this paper.
